# Risk for COVID-19 infection in patients with tobacco smoke-associated cancers of the upper and lower airway

**DOI:** 10.1007/s00405-020-06456-z

**Published:** 2020-11-20

**Authors:** Oreste Gallo

**Affiliations:** Department of Otorhinolaryngology, Careggi University Hospital, Largo Brambilla, 3, 50139 Florence, Italy

**Keywords:** COVID-19, Tobacco smoke, Interleukin-6, Head neck cancer, Lung cancer

## Abstract

**Purpose:**

Cancer patients are regarded as a group at risk for both COVID-19 infection and severe clinical course because of advanced age, comorbidities and iatrogenic immune impairment. Among them, patients with cancer of the upper and lower airways share other risk factors, mostly related to tobacco-smoke exposure, including male gender, airway epithelial damages, chronic obstructive respiratory disease (COPD), cardiovascular and cerebrovascular diseases. Clinical and pathophysiological factors shared by these conditions are reviewed.

**Methods:**

Review of the published literature since the beginning of 2020.

**Results:**

COVID-19 is a respiratory infectious disease and SARS-CoV-2 replication and shedding occurs in nasal and bronchial epithelial respiratory cells through the interaction with ACE2 and TMPRSS2 receptors, both overexpressed in smokers and former smokers. Tobacco-smoke airway exposure is also characterized by a chronic inflammation with activation of inflammatory cells and cytokine release including interleukin-6 (IL-6). A high release of cytokine in response to viral infection is documented in COVID-19 patients with adverse clinical outcomes and IL-6 is a key element of the cytokine storm syndrome leading to multi-organ damage.

**Conclusions:**

Patients with cancers of the upper and lower airways might be at increased risk of infection, morbidity and mortality from COVID-19 also because of tobacco exposure, a key factor in triggering inflammation, immunity and cancer.

## Introduction

COVID-19 is a coronavirus outbreak that initially appeared in Wuhan, Hubei Province, China, in December 2019, but it has already evolved into a pandemic spreading rapidly worldwide. As of 14 October 2020, a total number of 31 million cases of COVID-19 have been reported worldwide, including more than 1,000,000 deaths, the majority of which have been reported in Europe, USA, India and Brasil [1].

However, as the pandemic is still unfortunately under progression, few data are available regarding potential risk and prognostic factors in COVID-19 population [2]. Among them, smoking history has been correlated with a higher incidence and severity of SARS-CoV-2 infection [2, 3].

A Chinese study published in February 2020 based on a sample of 1.099 confirmed cases of COVID-19, comparing smokers and non-smokers, among 172 patients with severe symptoms, 16.9% were current smokers and 5.2% were former smokers, in contrast with non-complicated cases where 11.8% were current smokers and 1.3% were former smoker [2].

A higher risk of respiratory infection and mortality in smokers has been reported in previous studies during MERS-CoV outbreak, a coronavirus respiratory infection correlated with SARS-CoV-2, suggesting a detrimental effect of tobacco-smoke on host immune response [4].

Overall, a recently published systematic review on COVID-19 and tobacco-smoke reported in smokers the risk of severe symptoms 1.4 times higher (RR = 1.4, 95% CI 0.98–2.00), and the risk of intensive care unit admission, mechanical ventilation or death 2.4 times higher (RR = 2.4, 95% CI 1.43–4.04) than in non-smokers [3], further confirming a negative prognostic impact of tobacco-smoke in COVID-19 infected population.

A possible mechanism responsible for the worse impact of tobacco smoke in COVID-19 patients has been associated to the airway damage characteristic of former and current smokers as well as to smoke-related respiratory diseases including COPD [5]. Smoking is also detrimental to the immune system and its responsiveness to infections, making smokers more vulnerable to respiratory infectious diseases [5].

Since recent reports suggested a higher COVID-19 prevalence, morbidity and mortality in cancer patients [6, 7], patients with smoking related cancer of the upper and lower airways might represent a high-risk population for SARS-CoV-2 infection. Furthermore, because of advanced age, significant cardiovascular, cerebrovascular and respiratory co-morbidities, smoking-related airway damage, diabetes as well as the well-known treatment-related host immune impairment or suppression, they might be prone to severe outcome [5, 8]. To date, few reports have been published regarding patients with cancer of the airways suggesting a higher risk of infection by SARS-CoV-2, while no definitive data exist in relation to morbidity and mortality in this specific cancer population. The purpose of this study is to review current english literature about COVID-19 and tobacco-related cancers of the airways in order to better assess in these patients the complex interactions among tobacco-exposure, immune system, and prognosis when they are infected by SARS-Cov-2.

## The interplays between COVID-19, tobacco exposure, and tumours of the upper and lower airways

As in several infectious diseases, people with compromised immune system are at increased risk of COVID-19, which includes people with active cancer and many more cancer survivors. Among them, tobacco-smoke-related cancer population seems to be at higher risk [2, 3, 7]. While a possible correlation between lung cancer, tobacco smoke and higher risk of COVID-19 infection has been recently postulated in some early COVID-19 reports [6, 7], few data are available regarding the incidence of head and neck cancer (HNC) during SARS-CoV-2 infection. Therefore, HNC patients being heavy smokers and drinkers and showing immune system impairment, not only as consequence of cancer therapies, [9, 10] might represent a high-risk cancer population for COVID-19 infection.

In a respiratory infectious disease such as COVID-19, the airway represents the main source of viral loads. Accordingly, Zou et al. [11] showed higher viral loads after symptom onset, higher in the nose than in the throat, but SARS-CoV-2 has been also detected in lower respiratory tract samples [12]. In fact, although the nasal goblet and ciliated cells represent the main site of expression of ACE2 and TMPRSS2 receptors, both required for SARS-CoV-2 infection of host cancer cells [13], a recent study by Lukassen et al. [14] confirm that SARS-CoV-2 receptors are expressed also in bronchial secretory epithelial cells. Interestingly, a significant higher ACE2 expression has recently documented in bronchial epithelium from former and current smokers when compared with non-smokers [15]. Indeed, the goblet secretory nasal epithelial cells over-express ACE2 receptor, thus suggesting that the upper airway is the main initial target of SARS-CoV-2 involved in infection and transmission of the disease [16].

ACE2 protein does not work as a simple receptor, but is involved in activation of immune response by modulating cytokine releasing in the infected cell working as an interferon-stimulated gene [16]. After allowing SARS-CoV-2 virus internalization in airway epithelial cell, ACE2 protein can modulate the host interferon (IFN) response by promoting SARS-CoV-2 to maintain cellular targets in neighboring human upper airway epithelial cells. Whether IFN host activation is protective or detrimental to the host may depend on the stage of infection, viral clade, as well as other factors such as age, gender, individual immune reactivity and co-morbidities, possibly including cancer [16, 17].

Cancer development is usually associated with a blunted immune status characterized by over-expression of cytokines, altered release of pro-inflammatory signals, impaired dendritic cell activation and enhanced functional immunosuppressive lymphocytes populations [10]. Taken together, in cancer patients population, these features indicate a well-documented impairment of the immune system and immune responses with a potential higher risk of COVID-19 infection; on the other hand, COVID-19 patients with adverse clinical outcomes show an aggressive inflammatory response with dysregulation of host immune system characterized by the so-called cytokine storm [18].

Therefore, disease severity in COVID-19 patients is due not only to the viral infection but also to the host hyperinflammatory response. The high release of cytokines by the immune system in response to virus infection and/or secondary infections resulting in cytokine storm syndrome is responsible for uncontrolled systemic inflammation with multi-organ damage leading to organ failure, especially cardiac, hepatic and renal system and frequently to death [18]. Accordingly, many fatal cases of COVID-19 infection resulted in a high release of inflammatory cytokines, mainly of IL-6, and the latter is significantly elevated in patients with severe clinical course [19]. A recent meta-analysis of mean serum IL-6 concentrations reported 2.9-fold higher levels in patients with complicated COVID-19 infection when compared with patients without complications. For this reason, an humanized monoclonal antibody targeting IL-6 receptor, with the ultimate aim to block downstream pro-inflammatory effects of IL-6, named Tocilizumab, that was already approved by FDA for immune-mediated rheumatic disease, has been used in high-risk patients appearing efficacious and safe in preliminary investigations [19].

IL-6 is a pro-inflammatory cytokine involved in inflammation and in modulation of host immune response. At variance, IL-6 can also be released by various cells in the tumor microenvironment including the cancerous and stromal cells [20, 21]. Increased levels in serum and cancer tissue of IL-6 have been related to poor prognosis and lower survival rates in several human cancers (such as breast, renal, gastric, prostate, leukemia) [21–23], while down-regulation of IL-6 might be related to a better response to treatment representing a potential target for anticancer therapy [22]. IL-6 can also act on cancer cells in autocrine manner as a growth factor [24] and in paracrine fashion activating inflammatory surrounding cells [20, 21]. Furthermore, the pro- inflammatory IL-6 controls suppression of apoptosis by deleting the genes involved in cell cycle, inducing phosphorylation of STAT3 and STAT1 transcription factors [25].

Interestingly, we have documented a high serum level of IL-6 in HNC patients when compared to healthy controls and IL-6 levels correlated with acute-phase proteins in HNC, suggesting its central role in controlling inflammatory responses in HNC patients [26]. More recently, similar results have been reported in large series of non-small lung cancer being associated with tumor stage and a worse prognosis [27, 28]. Moreover, IL-6 is highly expressed in male smokers in non-small lung cancer patients and in HNC with an unfavourable prognostic significance [29, 30].

The cigarette-smoke is associated with a generalized airway inflammation and it is likely that IL-6 plays a role in the inflammatory response associated with smoking. Thus, IL-6 is possibly involved in alterations of immune responses in the tobacco-smoke airways triggering chronic inflammation capable to induce cell transformation and subsequent tumor growth as well as being involved in allergy, asthma and other pulmonary diseases [31].

It is well known that inflammatory responses might play a dual role in tumor development, supporting the inhibition of tumor growth by promoting the anti- tumor activity of cytotoxic T cell [20, 28]; on the other hand, the induction of DNA damage by free radical production in chronic inflammation can contribute to tumorigenesis and progression [20, 32]. Taken together, it is likely that in tobacco-related cancer of the airways, the respiratory mucosal inflammation characterized by high IL-6 and cytokines release, frequently associated to cancer growth and progression, may contribute to COVID-19 infection and severity because of a pre-existent unbalanced host immune response.

To date, few case series of cancer patients infected by COVID-19 have been published in the current English literature, most of these without reporting the index cancer. Therefore, current evidences remain insufficient to draw a conclusive association between cancer and COVID-19 infection. Indeed, a specific meta-analysis pooling data from the recent literature seems to confirm that patients with cancer and cancer survivors represent an important at-risk population for COVID-19 [33]. Accordingly, Liang et al. [6] reported a cancer prevalence of 1% (95% CI 0.61–1.65%) among the 1590 patient cases of COVID-19. They found a higher risk of severe events compare with those without cancer in terms of 39 versus 8%, (HR:5.34; CI 1.80–16.18; *P* = 0.0026), however, the significance of this report is limited by the only 18 cancer patients examined overall. Among them, four (22.3%) had lung cancers, while no one was affected by HNC. At variance, in a paper by Zhang et al. [7] regarding 28 COVID-19-infected patients with active cancer, they reported four esophageal and two laryngo-pharyngeal squamous cell carcinomas (21.5%), together with seven lung cancer patients, accounting for 13 out of 28 cases (46.5%) described. Additional reports seem to confirm a higher risk of COVID-19 infection in cancer patients and smokers [34, 35], however, to date, no definitive evidence can be drawn.

More recently, in an extensive meta-analysis evaluating 32 studies involving 46,499 COVID-19 patients from Asia, Europa and U.S.A, of whom 1776 with cancer Giannakoulis [36] reported that cancer from several districts is associated with worse clinical outcomes in case of SARS-CoV-2 infection. However, elderly patients with cancer may be not at increased risk of death when infected with COVID-19.

Furthermore, Lee et al. [37] in a prospective cohort study enrolling patients with cancer in the UK, documented that patients with different cancer types have different susceptibility to SARS-CoV-2 infection and COVID-19 phenotypes when compared with a parallel non-COVID-19 UK cancer control population. A critical factor also in cancer population with SARS-CoV-2 infection was advanced age and male gender with the all-cause case-fatality rate rising from 0.10 in patients aged 40–49 years to 0.48 in those aged 80 years and older. According to cancer subtype, patients with haemathological malignancies (leukemia, lymphoma, and myeloma) showed an increased susceptibility to be infected by SARS-CoV-2 and a higher risk of severe evolution together with old male population affected by prostate cancer. However, in this series, COVID-19 patients with cancers of the upper or lower airway did not show an increased risk of mortality when compared with non-COVID-19 controls.

We have recently revised our series of 120 consecutive hospitalized COVID-19 patients with mild/moderate disease, including 18 cancer patients (12 male, with a median age of 73.7 vs. 64.2 years in non-cancer population, *p* = 0.018) [38]. Overall, 33.3% in the cancer group died from COVID-19, while among non-cancer patients, fatal evolution was reported in a lower yet not statistically significant rate (13.9%; *p* = 0.289). By univariate logistic regression analysis, cancer population seemed to have an increased risk of death (OR = 4.77, *p* = 0.0013); however, when performing multivariate analysis accounting for age, smoking status and cardiovascular disease, the result was no longer significant.

Among them, we reported two cases of HNC patients, another was hospitalized after the publication of the aforementioned article. They included two laryngectomized patients, the first with a T4N1M0 squamous cell carcinoma of the larynx and the latter with an hypopharyngeal T4N2M0 disease [39]. Interestingly, despite the upper airways are functionally excluded from the lungs after total laryngectomy, both patients resulted positive by RT-PCR test for SARS-CoV-2 RNA in both nasopharyngeal and tracheal samples. Because of surgery, the lack of a functional nose able to filter, humidify, and warm inhaled air, with a possible direct inhalation of infected droplet and aerosol into the lungs, together with a higher ACE2 expression on epithelial lower respiratory cells, due to former cigarette-smoking exposure, might justify the higher risk of SARS-CoV-2 infection with an early severe pneumonia documented by us and others in this specific cancer population.

How SARS-CoV-2 is able to infect non-functional upper airway is intriguing. Total laryngectomy, by excluding nasal breathing, inevitably results in a loss (or at least a serious decrease) of the sense of smell and taste. In fact, only a minority of laryngectomized patients maintain at least in part olfactory function, allowing odorant molecules to reach and stimulate olfactory mucosa through the nose or nasopharynx [40]. However, the mechanism(s) by which odorant molecules reach olfactory mucosa is not completely understood.

The development of a specific technique for olfaction rehabilitation using a nasal-airflow inducing maneuver after total laryngectomy suggests a possible passage of air through the nose able to stimulate olfaction, which in turn might be a possible route for the SARS-CoV-2 infection of the upper airway. Two main hypotheses were formulated suggesting a retronasal or an anteronasal air flow in laryngectomized patients. The generation of a negative pressure (“underpressure”) in the oral cavity by an extended yawning movement (“polite yawning maneuver”) [40] generates an airflow through the nose, thus potentially stimulating the olfaction in a physiologic manner. Because of a correlation between the quality of the esophageal voice and olfaction acuity of the laryngectomized patients, (probably as a result of the better control of oropharyngeal musculature in good esophageal speakers, enabling them to pump air into the nasal cavity), a retronasal airflow has been postulated. Conversely, others suggest an anteronasal flow of the air as a consequence of the oropharyngeal movements improved by polite yawning maneuver [40, 41].

**Fig. 1:**
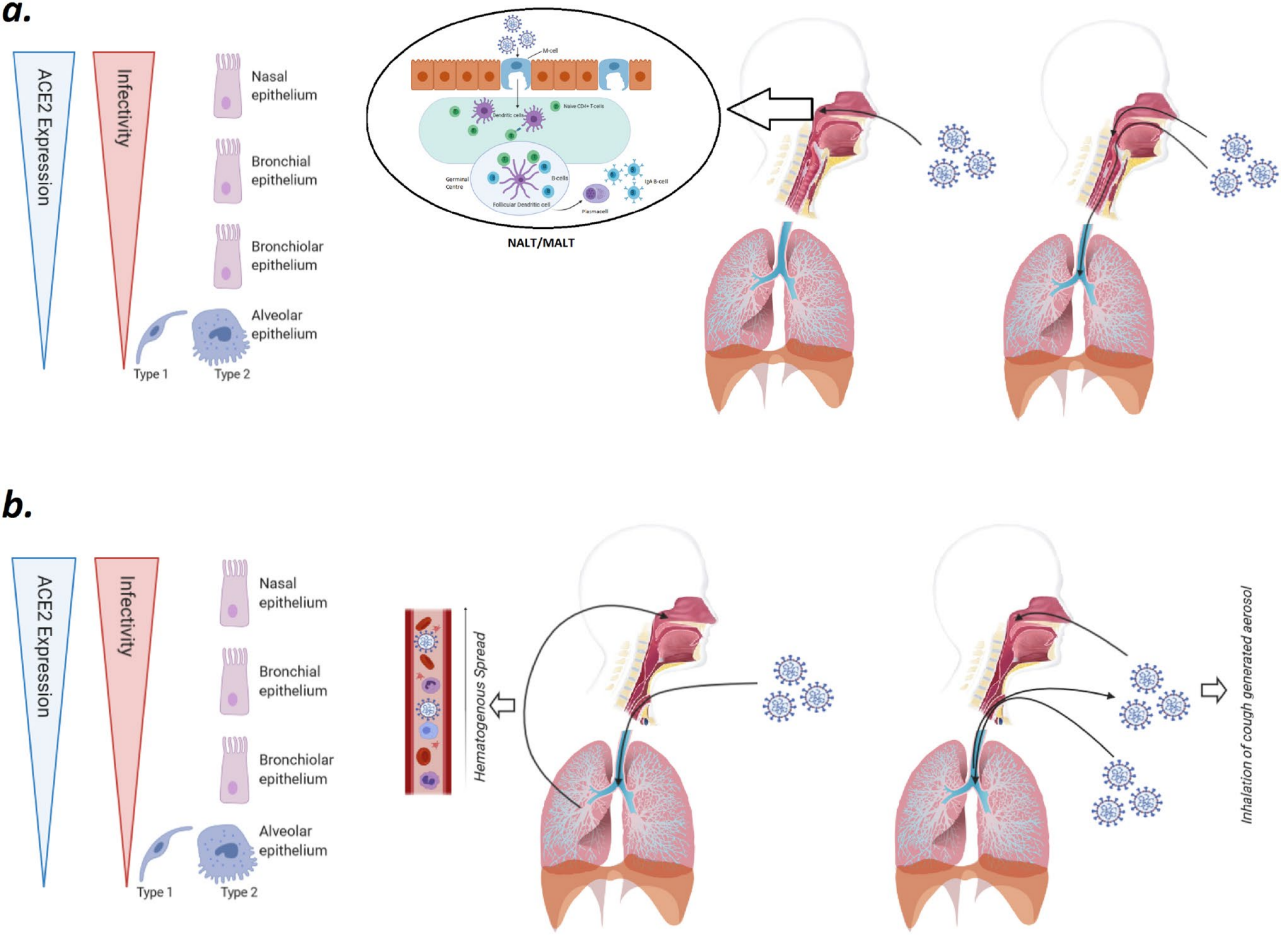
**a** ACE2 expression, infectivity in upper and lower airway in a normal subject. **b** Possible SARS-CoV-2 spread routes in a laryngectomized patient

Therefore, it is likely that also in COVID-19 laryngectomized patients, the virus can reach the anatomically excluded upper airway from the trapped air into the oral cavity or alternatively from the infected tracheobronchial secretions, aerosolized during coughing or talking using esophageal voice with and without prosthesis [42] (Fig. 1).

An alternative hypothesis suggests that once the virus has been inhaled through the tracheostoma reaching deep lung tissues with pneumonia, a haematogenous spread might be responsible for SARS-CoV-2 colonization of the upper airway. This possibility has been postulated in an elegant study by Hou et al. [43] evaluating the infectivity of the SARS-CoV-2 also in relation to ACE2 expression in the airways. The study reveals the highest ACE2 expression in the nose with decreasing expression throughout the lower respiratory tract, paralleled a striking gradient of SARS-CoV2 infection from the nose to the lung (Fig. 1). Accordingly, the haematogenous spread of the virus has been clearly documented by systemic endotheliitis with multiorgan failure described in severe COVID-19 infection [44] as well as in COVID-19 patients with severe neurological complications [45].

The apparently innocent nasal localization in these patients must not go unnoticed: in a bidirectional unified airway model, the type of local immune response in the nasal mucosa (the so-called nasopharyngeal-associated lymphoid tissue, NALT) has the chance to drive the systemic cytokine response and the priming of the lung immunity [46, 47]. In addition, there is experimental evidence that nasal exposure to non-SARS-CoV-2 coronavirus is capable to prepare the lower airways for a faster and enhanced innate and adaptive response to the pathogen, with a final overall reduction in terms of both morbidity and mortality in infected mice [47].

Among the three HNC patients, all were admitted in Intensive Care Unit and early intubated for mechanical ventilation, while two died from COVID-19 (66.6%).

A higher risk in HNC patients who underwent a total laryngectomy has been recently also reported by others [48], confirming that patients with iatrogenic anatomic exclusion of the upper airway might represent a specific high-risk population for COVID-19 infection. Overall, these preliminary results confirm that HNC, in particular after a total laryngectomy, has to be regarded as at risk of COVID-19 infection with severe evolution [49].

Among patients with thoracic malignancies, including non-small-cell lung cancer (NSCLC), recently a registry-based series from the Thoracic Cancer International COVID-19 Collaboration (TERAVOLT) group reported among 200 COVID-19 infected patients with cancer a higher incidence of NSCLC (151 of 200, 72%) and current or former smokers (159 of 196, 81%). Of these, 152 (76%) were hospitalized and 66 (33%) died from COVID-19. In univariate analysis, smoking history, age and current chemotherapy were associated with an increased risk of death. However, at multivariate analysis, only smoking history maintained its prognostic impact (OR = 3.18, 95% CI 1.11–9.06). To date, this represents the larger series of COVID-19 patients with active lower airway malignancies published in the English literature [50].

In the current literature, conflicting results about a higher mortality rate in COVID-19-infected cancer population have been reported [6, 7, 36–38]. Together with studies suggesting a generalized increased mortality in cancer population because of the systemic immunesuppressive status caused by malignant disease or anticancer treatment, others reported a higher mortality rate only for some primary tumour subtype, not including SCLG and HNC, and for patients who had recent chemotherapy [37].

## Concluding Remarks 

The airways represent the main port of entry of all respiratory infections including COVID-19. SARS-CoV-2 is able to infect respiratory epithelial cells that show ACE2/TMPRSS2 receptors highly expressed in the mucosa of the upper and lower airways. Accordingly, in symptomatic cases, COVID-19 infection is characterized by specific upper and lower respiratory symptoms, including anosmia, dysgeusia, sore throat, cough, and shortness of breath [2].

A key role of the upper airway has been recently documented by elegant studies investigating the tropism and the mechanism of immune response against SARS-CoV-2 in the nasal cells [13]. As many other respiratory viral diseases, it is believed that penetration into the upper airways is the first step of the infection as higher viral load was found in nasal swabs when compared to throat swabs in COVID-19 patients [11]. By single-cell RNA sequencing, nasal goblet and ciliated epithelial cells were shown to have the highest levels of ACE2 expression in vitro as well as the highest infectivity in vivo when compared with other respiratory tracts, thus suggesting that the nasal mucosa may be the dominant initial site for SARS-CoV-2 respiratory tract infection [47]. From experimental evidence, the nasal mucosa appears to be both a target and a key factor in the regulation of the in vivo immune response which seems to be mainly driven by IFN-alpha stimulated pathway. As previously stated, NALT is considered to regulate the mucosal immune response in the nasal mucosa and upper respiratory tract [46, 47].

Furthermore, recent data suggest that an imbalanced host response to SARS-CoV-2 is responsible for development of COVID-19 infection and severe evolution [51]. COVID-19 infection seems to be determined by a low levels of Type I and III interferons juxtaposed to elevated chemokine release and high expression of IL-6 in respiratory cell lines. Therefore, a reduced-innate antiviral defense coupled with exaggerated inflammatory IL-6 production might be responsible for SARS-CoV2 airway infection and dissemination as well as for the cytokine storm associated to worse progression in COVID-19 patients [51].

In this context, nasal secretory and ciliated cells seem to have key role suggesting that the upper respiratory tract is the viral reservoir and site of active replication [11, 17, 47].

The nasal mucosa and its associated lymphoid-tissue (NALT) is an ancient organ which has evolved along with olfactory function in the vertebrates and the relationships between allergic and other immune-mediated conditions of the upper airways and the role of viruses are very complex [52–55]. Together, a cross-talk between upper and lower airways seems to be the key factor in the transmission of infection from the early site of virus replication to the lungs and this transmission seems to be regulated by activation of innate immunity by NALT with a modulation of the immune response at lower respiratory tract [46, 52, 53].

Transnasal exposure to pathogens is being clinically exploited to deliver vaccines (e.g., against the Influenza virus) even though the mechanisms underlying the generation of nasal antigen-specific tissue-resident memory T cells (TRM) are not well understood [56, 57]. More recently, a group of researchers have exploited intranasal administration of a chimpanzee Adenovirus (simian Ad-36)-based SARS-CoV-2 vaccine (ChAd-SARS-CoV-2-S) in a mouse model [58]. Strikingly, intranasal ChAd-SARS-CoV-2-S induces mucosal immunity, provides superior protection compared to the parenteral route, preventing SARS-CoV-2 lung infection and pneumonia in mice and it can even promote sterilizing immunity that can block interhuman transmission [58].

Taken together, these findings further support the concept of “united airways” suggesting not only a common and strictly correlated immune response to airborne pathogens among upper and lower respiratory tracts but also a common or at least a comparable response to carcinogens (tobacco smoke) as well to SARS-CoV-2 infection.

Finally, it is likely that factors affecting airway health status such as cigarette smoking or respiratory allergy can play a role in COVID-19 infection and progression. In fact, smokers and former smokers are more prone to be infected by SARS-CoV-2 with a higher risk of severe complications [2, 3].

The smoke-related over-expression of ACE2/TMPRRS2 receptors supports this hypothesis and patients with smoking-related cancers of the airways might represent an important at-risk cancer population. Indeed, in COVID-19 fatal cases, a multi-organs aggressive disease has been reported, involving not only the airways although they are always involved.

The uncontrolled inflammatory host response against virus aggression, starting from airways colonization and infection, can produce a cytokine storm syndrome and IL-6 has been indicated as a key pathogenetic factor, thus representing a promising target of experimental biologic therapies in severe COVID-19 cases.

IL-6 and other immune mediators might play a dual role in the pathogenesis of COVID-19. Host immune response is crucial to fight viral infections including SARSCoV-2, however, if COVID-19 activates an aggressive inflammatory response, this can result in damage of lungs and other tissues. In severe cases, the uncontrolled inflammation can produce a vast release of inflammatory cytokines, responsible for the multi-organ damage and related organ failure [18]. Despite an altered host immune reactivity is frequently reported in cancer population, the increased IL-6 production by inflammatory and airway epithelial cells of smokers might be actively involved in the pathogenesis of such an aggressive immune response. Thus, the tobacco-induced chronic inflammation can damage epithelial airway cells inducing ACE2 overexpression with an increased IL-6 release and a reduced production of IFN responsible for host defense response against viruses [51]. The high circulating level of IL-6 released in COVID-19-infected patients with smoking-related cancers might indicate an host immune inbalance, resulting in an exaggerated immune response and hyperinflammation with a cytokine storm syndrome and severe evolution. Therefore, it is possible to hypothesize that the airway damage by heavy tobacco-smoke exposure acts both in transforming epithelial cells and generating a heavy chronic inflammation of the upper and lower respiratory tract with an host inflammatory response characterized by a high release of cytokines, mainly IL-6, thus exposing active and former smokers to cancer of the airways as well as to SARS-CoV-2 severe infection.

In conclusion, the findings reported in this paper strongly suggest that patients with smoking-related cancers of the upper and lower airway represent a specific population with common epidemiology and co-morbidities and they should be considered at high risk of COVID-19 infection with severe prognosis.
